# Supplementary material for: A machine learning algorithm with subclonal sensitivity reveals widespread pan-cancer human leukocyte antigen loss of heterozygosity
Source: Nat Commun. 2022 Apr 12;13:1925. doi: 10.1038/s41467-022-29203-w (PMC9005524; doi:10.1038/s41467-022-29203-w)
Supplement: Supplementary file 1 — Supplementary Information [file 41467_2022_29203_MOESM1_ESM.docx]

# Supplementary Information for

**A machine learning algorithm with sub-clonal sensitivity reveals widespread pan-cancer HLA LOH**

Rachel Marty Pyke^1^, Dattatreya Mellacheruvu^1^, Steven Dea^1^, Charles W. Abbott^1^, Lee McDaniel^1^, Devayani P. Bhave^1^, Simo V. Zhang^1^, Eric Levy^1^, Gabor Bartha^1^, John West^1^, Michael P. Snyder^2^, Richard O. Chen^1,^ * and Sean Michael Boyle^1,^ *

^1^Personalis, Inc., Menlo Park, CA; ^2^ Stanford University, Palo Alto, CA

* These authors jointly supervised this work.

Co-corresponding authors and emails: Sean Michael Boyle ([sean.boyle@personalis.com](mailto:sean.boyle@personalis.com)) and Richard O. Chen ([richard.chen@personalis.com](mailto:richard.chen@personalis.com)).

**This PDF file includes:**

Supplementary Figures 1-18

Supplementary Tables 1-4

**Supplementary Figure 1.**

**
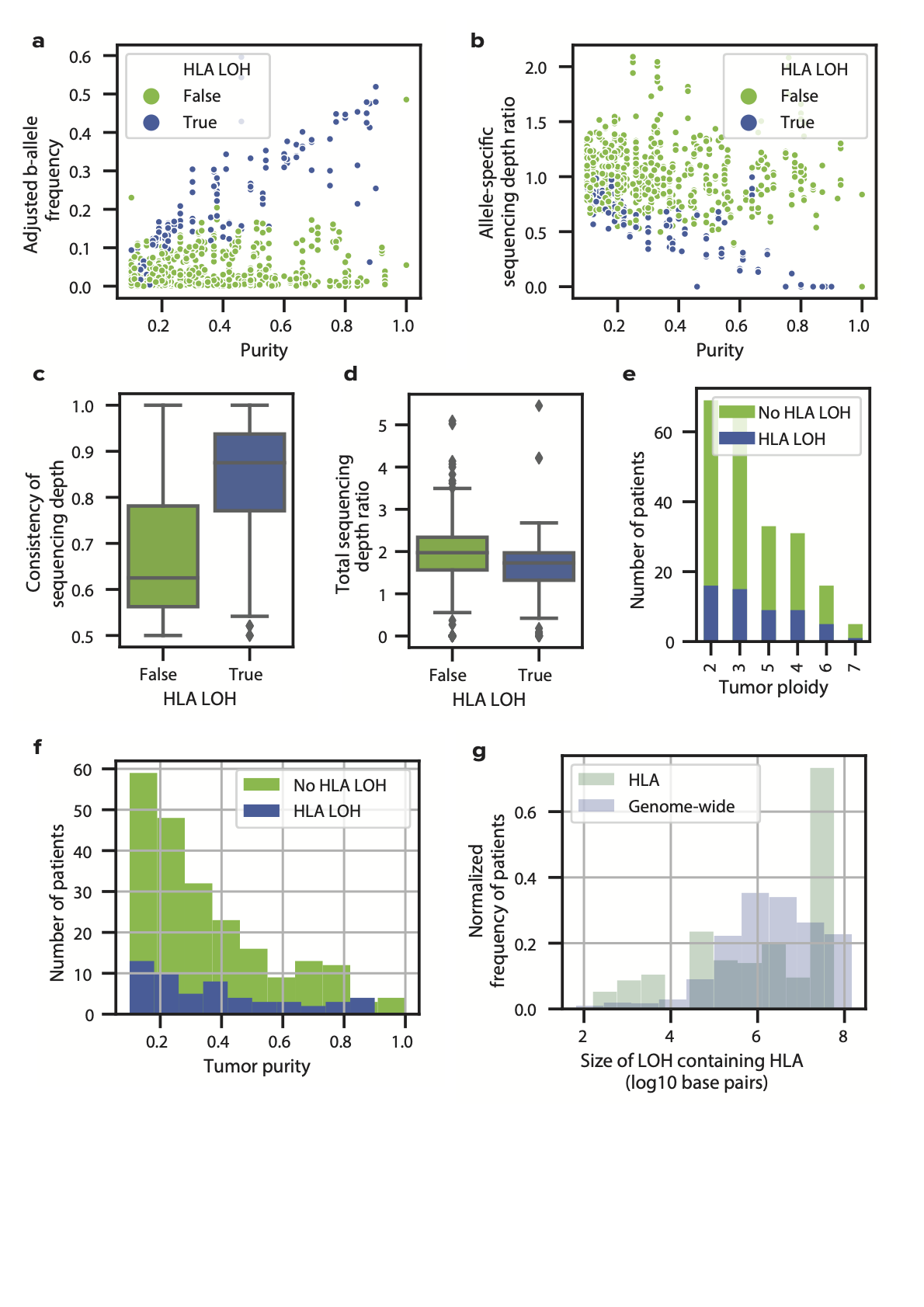
**

Overview of features in training data set. (A) A scatter plot showing the relationship between Adjusted B-allele Frequency and tumor purity (n=720 heterozygous genes). Genes with HLA LOH are shown in blue and genes without HLA LOH are shown in green. (B) A scatter plot showing the relationship between allele-specific sequencing depth ratio and tumor purity (n=720 heterozygous genes). Genes with HLA LOH are shown in blue and genes without HLA LOH are shown in green. (C-D) Boxplots showing the difference in distribution between (C) consistency of sequencing depth and (D) total sequencing depth for patients with and without HLA LOH for n=720 heterozygous genes. The center of the box denotes the median value, the box denotes the quartiles and the whiskers denote the remainder of the distribution apart from outliers. (E-F) Histograms showing the distributions of tumor (E) ploidy and (F) purity for patients with and without LOH (n=267 patients). Genes with HLA LOH are shown in blue and genes without HLA LOH are shown in green. (G) A histogram showing the distribution of LOH sizes that contain HLA genes across all patients (n=205 HLA deletions, 27534 genome-wide deletions from 610 patients). Source data are provided with this paper.

**Supplementary Figure 2.**


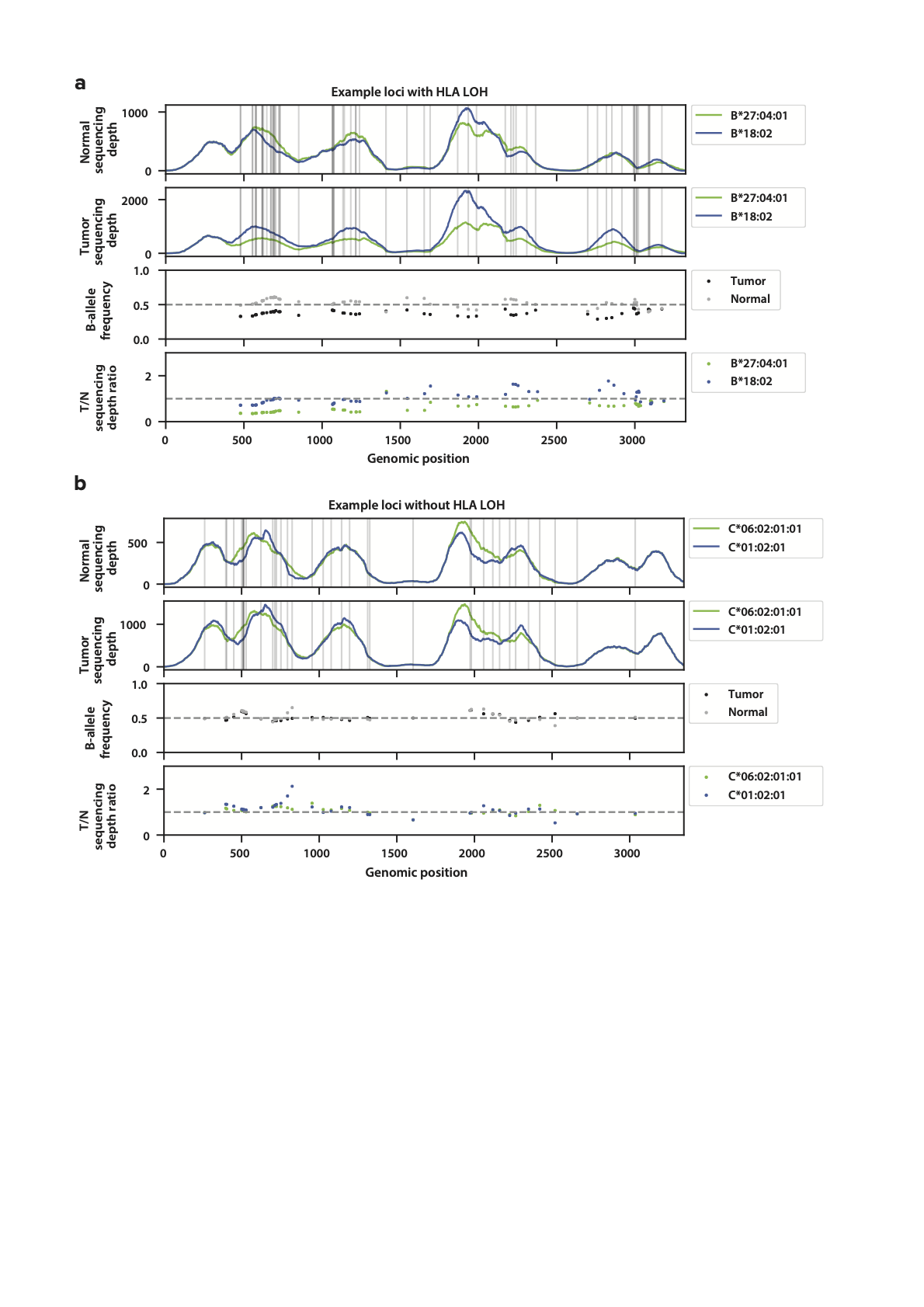


Overview of features in manual annotations. (A) Example of the feature output of a locus containing a manually annotated HLA LOH event. Blue and green denote the different alleles for a specific locus for normal sequencing depth, tumor sequencing depth, and tumor / normal (T/N) sequencing depth ratio. Black and white denote the tumor or normal B-allele frequency respectively. Normal sequencing depth, Tumor sequencing depth, B-allele frequency, and T/N sequencing depth ratio were all used to determine if a sample was manually annotated with HLA LOH. (B) Example of features of a locus not containing a manually annotated HLA LOH event. Source data are provided with this paper.

**Supplementary Figure 3.**


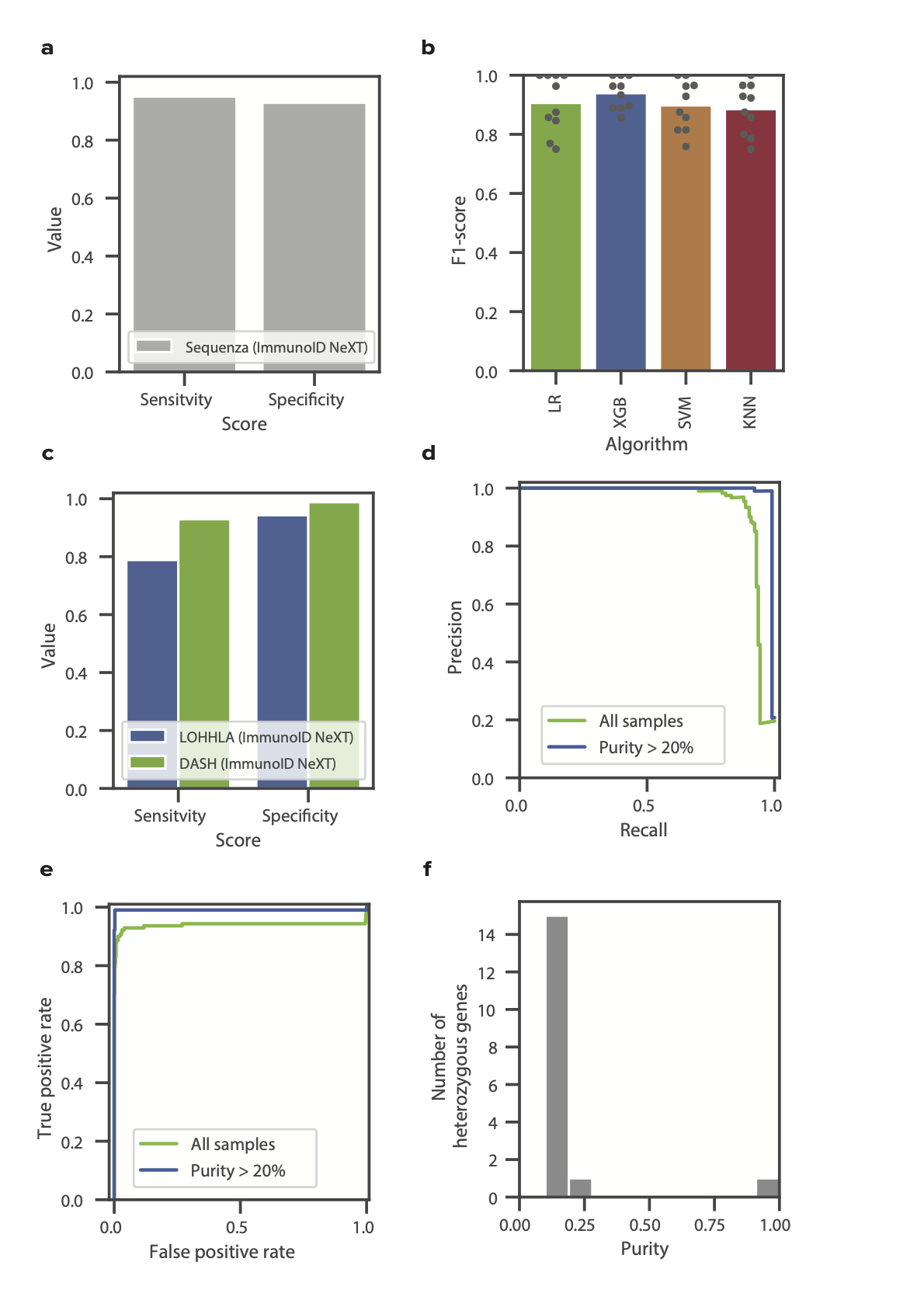


Performance of DASH. (A) Bar plot showing the sensitivity and specificity of using Sequenza to detect HLA LOH in ImmunoID NeXT samples (n=720 heterozygous genes). (B) Bar plot of F1-scores of different classifier machine learning algorithms using a 10-fold cross validation method. Dots represent the performance of the 10 individual algorithms (n=720 heterozygous genes across all folds). Abbreviations are as follows: LR - linear regression, XGB - XGBoost, SVM - support vector machine, KNN - K nearest neighbor. (C) Bar plots showing the sensitivity and specificity scores across ImmunoID NeXT samples between LOHHLA (blue) and DASH (green) (n=715 heterozygous genes; 5 are excluded from the full 720 genes as described in the methods). (D) Precision-recall curve for DASH across all samples in green and all samples with a tumor purity > 20% in blue (n=720 heterozygous genes). (E) ROC-curve showing the relationship between true positives and false positives for DASH across all samples in green and all samples with a tumor purity > 20% in blue (n=720 heterozygous genes). (F) A histogram showing the distribution of tumor purity values for alleles that were predicted incorrectly by DASH (n=17 heterozygous genes). Source data are provided with this paper.

**Supplementary Figure 4.**

**
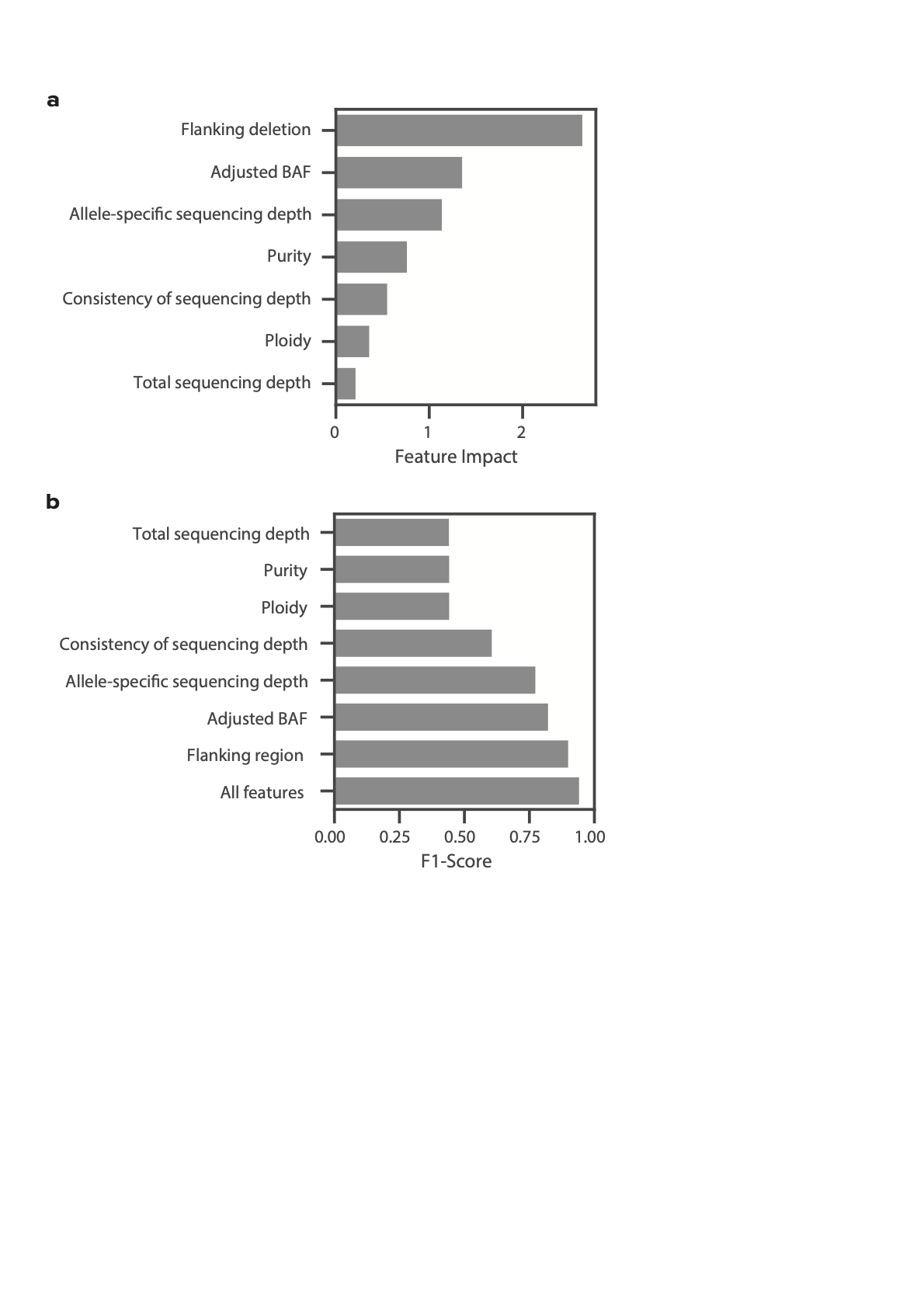
**

DASH feature impact. (A) Bar plot quantifying the impact an individual feature has on the DASH algorithm. Higher feature impact denotes a feature having a higher weight in the algorithm. (B) Bar plot displaying the F1-score of DASH trained with only the corresponding feature. Source data are provided with this paper.

**Supplementary Figure 5.**


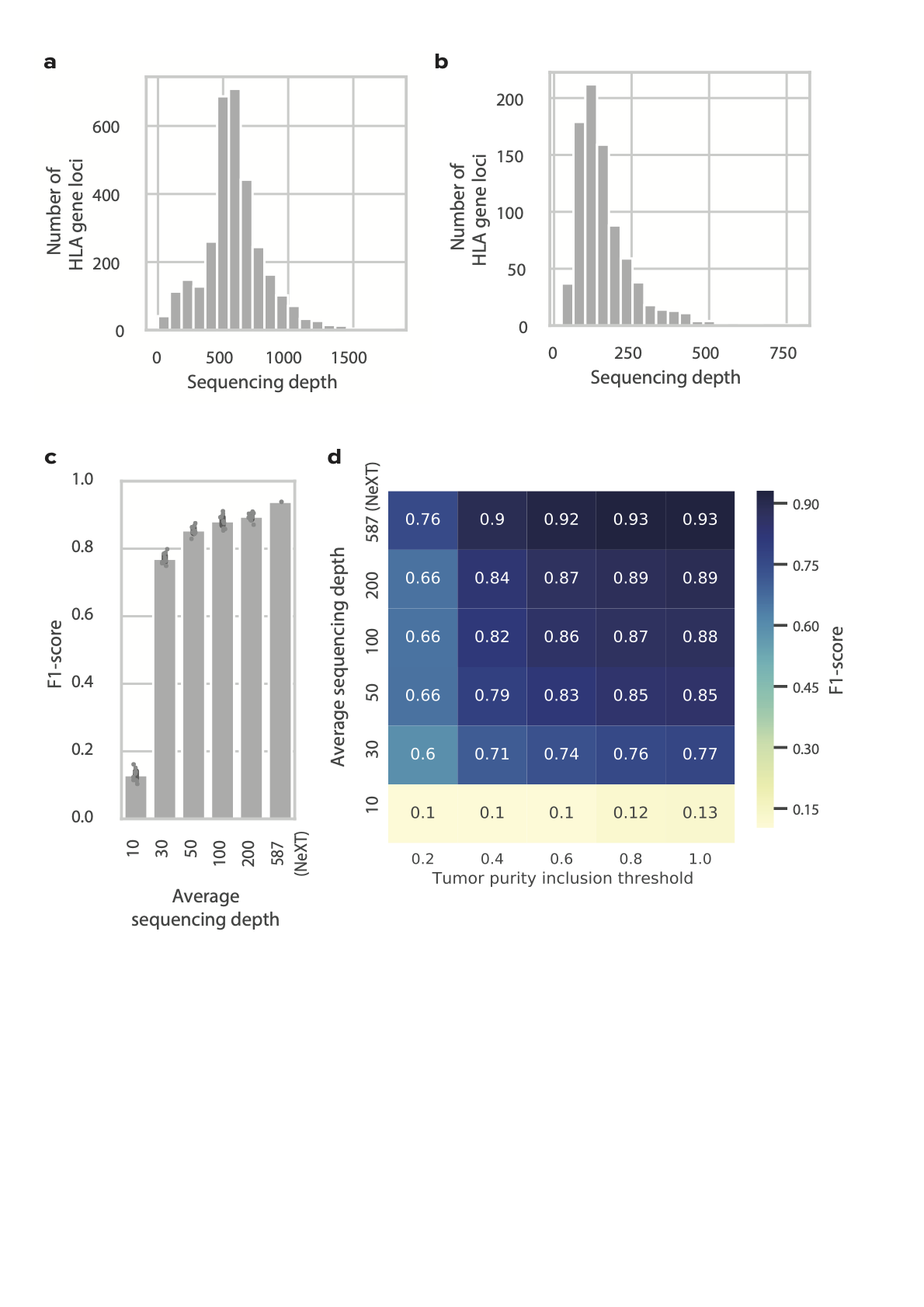


Performance of DASH with reduced sequencing depth. (A-B) Histograms displaying the average sequencing depth found in the HLA gene loci in (A) ImmunoID NeXT and (B) standard exome sequencing. (C) Bar plot of the average F1-scores when DASH was run on the same set of samples across different sequencing depths with n=10 subsampling replicates. The average sequencing depth corresponding to 587 represents the average sequencing depth found in ImmunoID NeXT samples. 95% confidence intervals are shown in dark gray. (D) Heatmap of the average F1-scores when DASH was run on the same set of samples across different sequencing depths and split by the sample’s tumor purity inclusion threshold. Source data are provided with this paper.

**Supplementary Figure 6.**

**
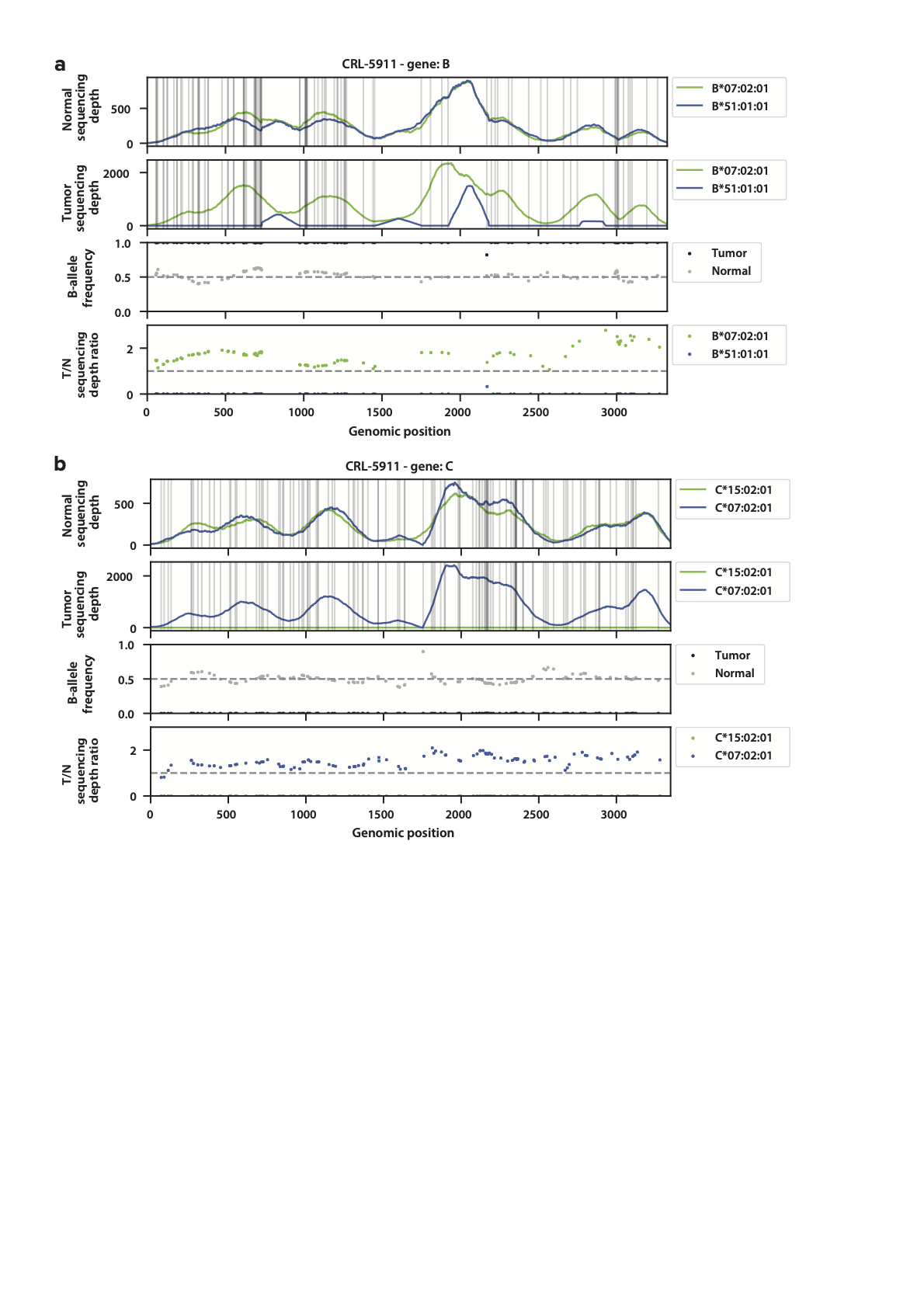
**

Visualization of the CRL-5911 cell line with HLA LOH. (A-B) Overview of features that show HLA LOH in (A) *HLA-B* and (B) *HLA-C* of the CRL-5911 cell line. Source data are provided with this paper.

**Supplementary Figure 7.**

**
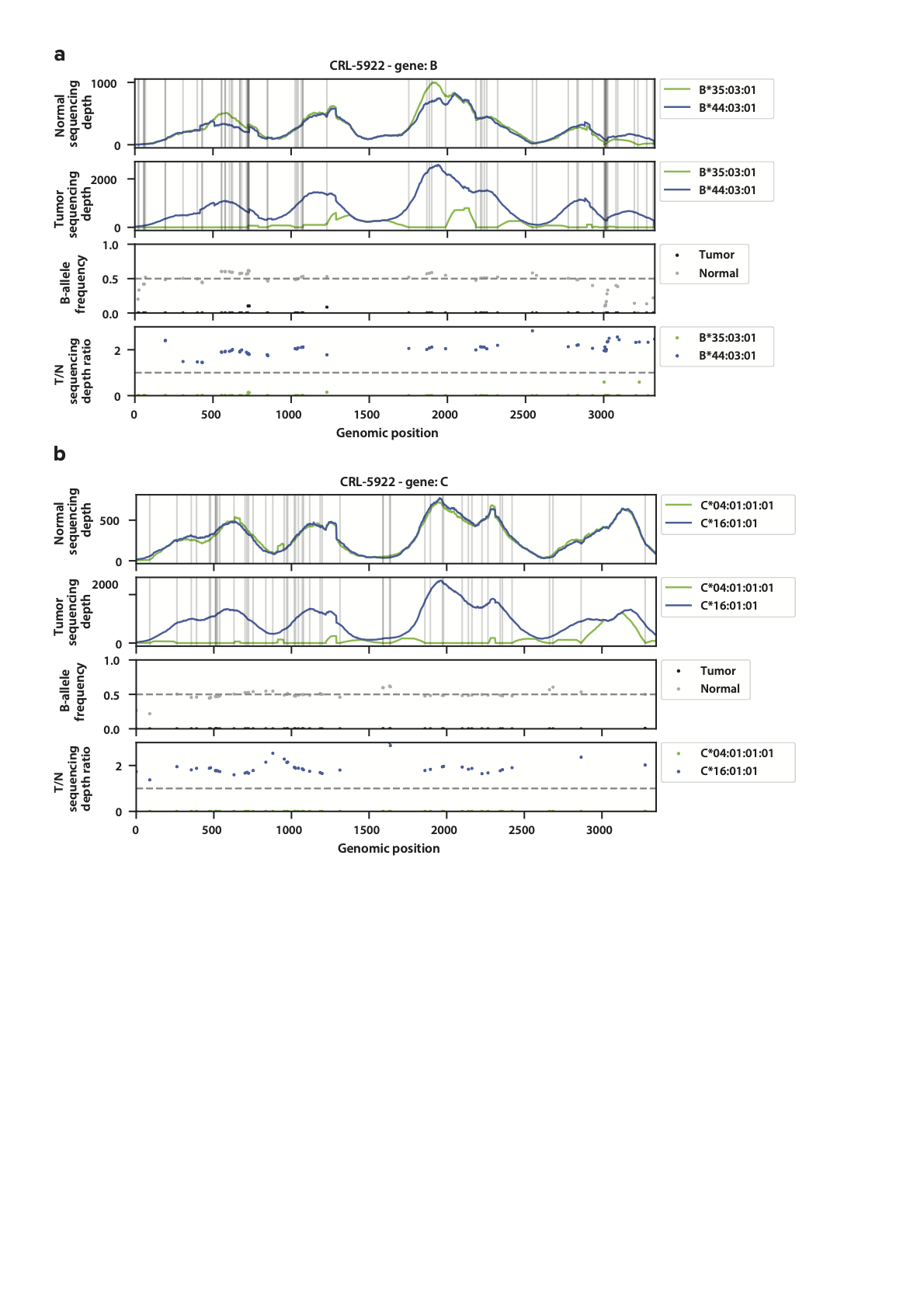
**

Visualization of the CRL-5922 cell line with HLA LOH. (A-B) Overview of features that show HLA LOH in (A) *HLA-B* and (B) *HLA-C* of the CRL-5922 cell line. Source data are provided with this paper.

**Supplementary Figure 8.**

**
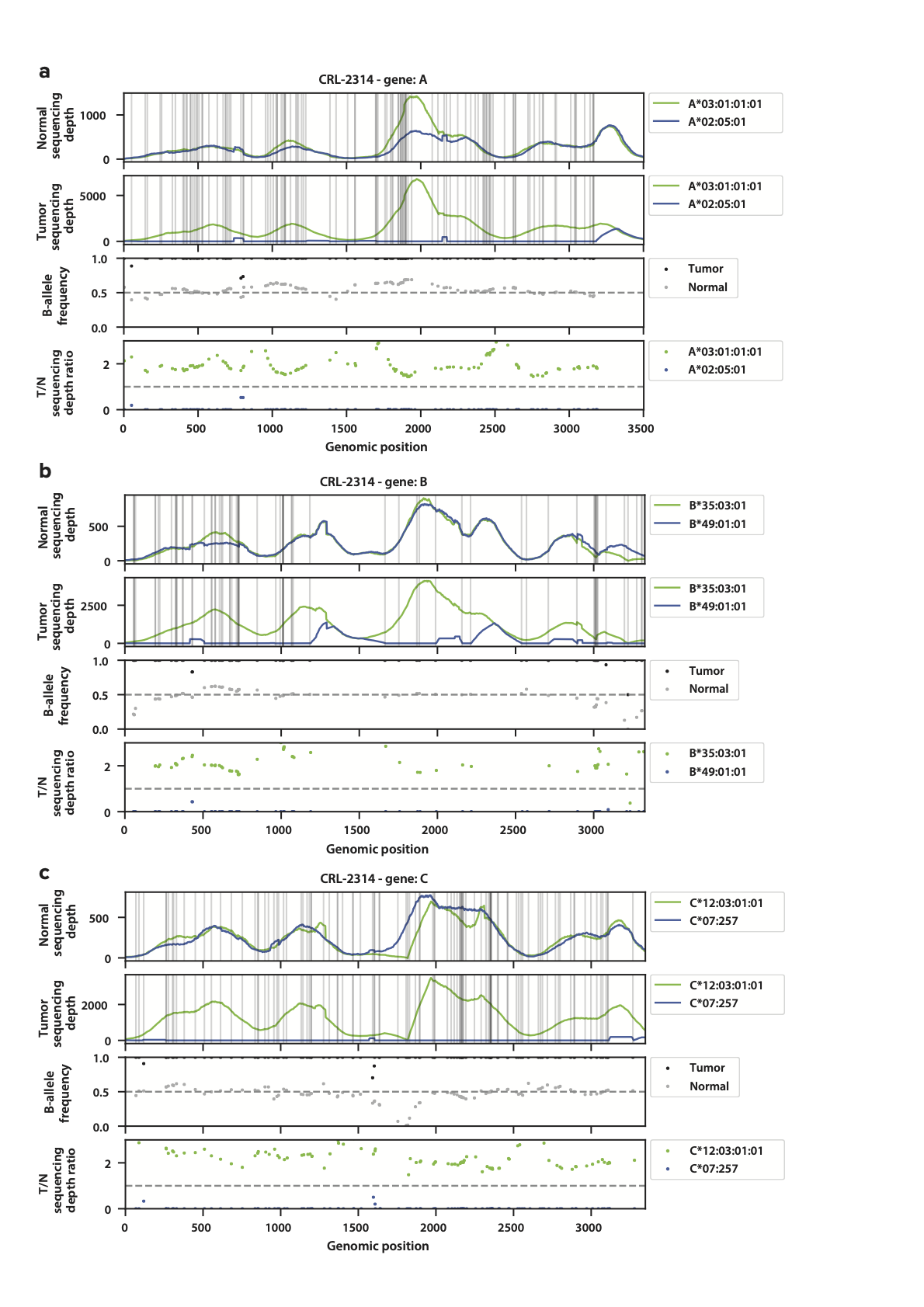
**

Visualization of the CRL-2314 cell line with HLA LOH. (A-B) Overview of features that show HLA LOH in (A) *HLA-A*, (B) *HLA-B*, and (C) *HLA-C* of the CRL-2314 cell line. Source data are provided with this paper.

**Supplementary Figure 9.**
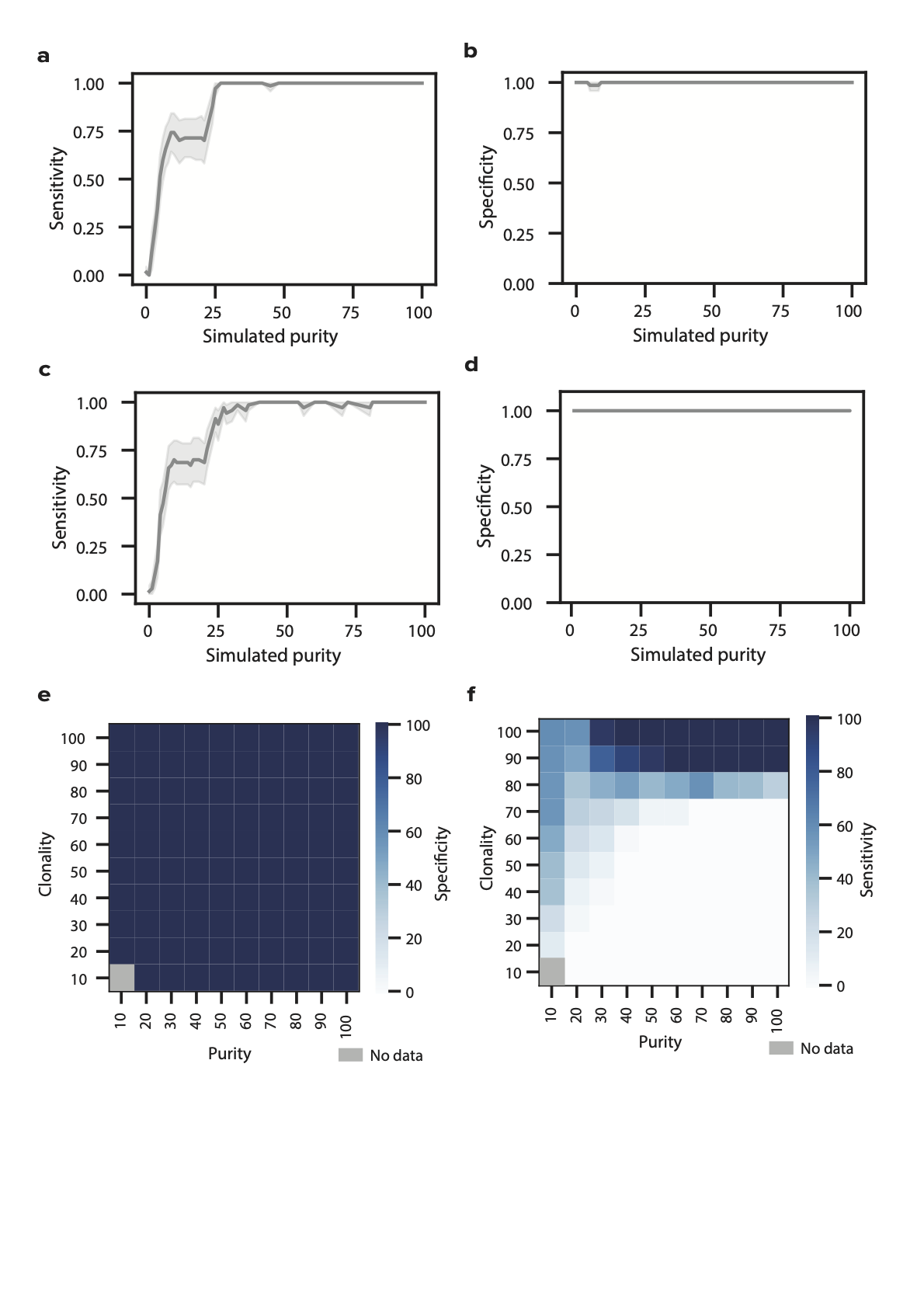


Combined DASH and LOHHLA performance on three cell lines. (A-B) Line plots showing the measured (A) sensitivity and (B) specificity of DASH and the measured (C) sensitivity and (D) specificity of LOHHLA at various purity levels with fully clonal tumors. Shaded region denotes 95% confidence intervals. (E-F) Heatmaps showing the (E) specificity and (F) sensitivity of LOHHLA to capture HLA LOH in simulated samples of differing purity and clonality. Dark blue denotes high sensitivity or specificity, light blue denotes low sensitivity or specificity and gray denotes no data. Source data are provided with this paper.

**Supplementary Figure 10.**


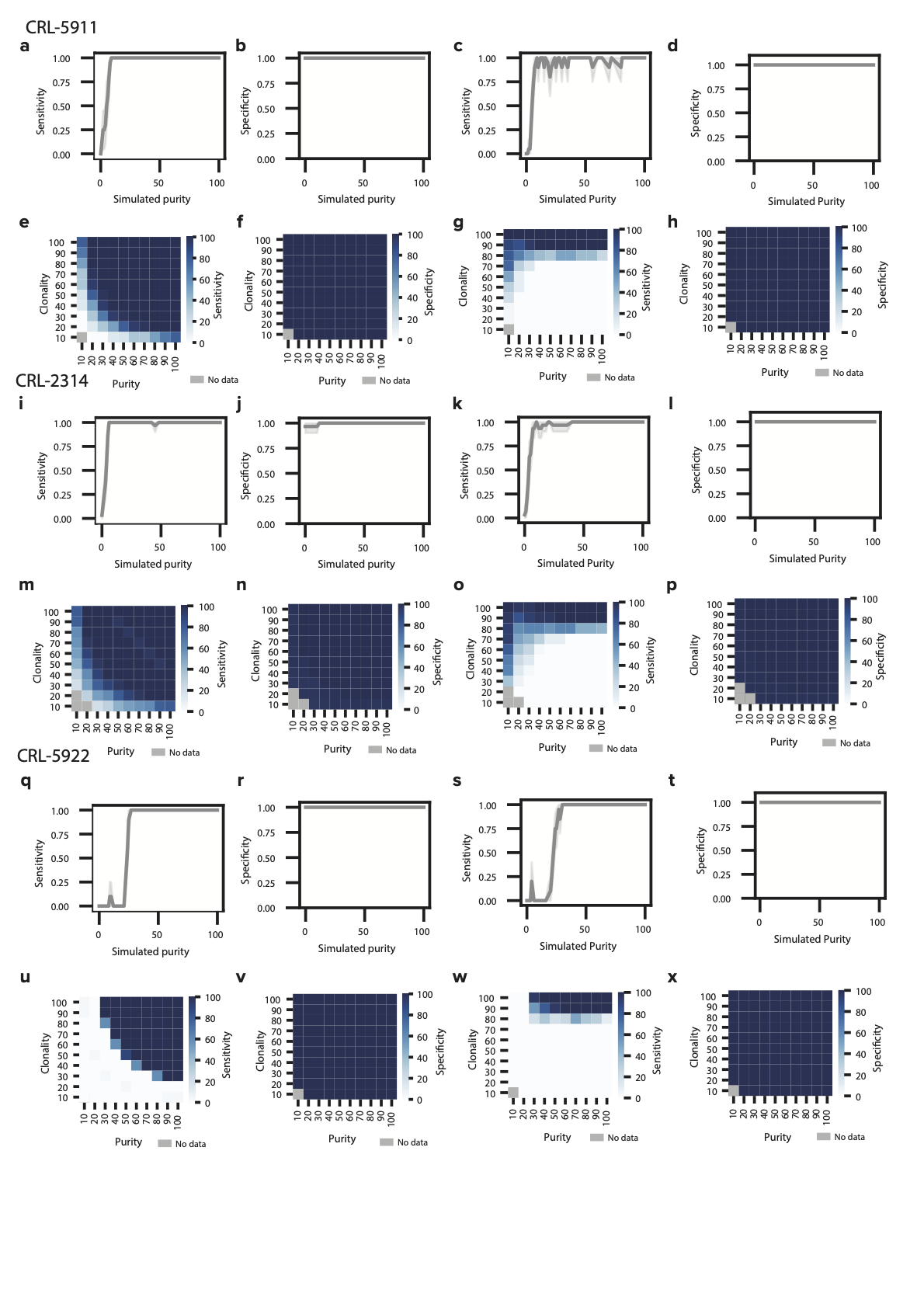


Individual DASH and LOHHLA performance on three cell lines. (A-B, I-J, Q-R) Line plots showing the measured (A, I, Q) sensitivity and (B, J, R) specificity at various purity levels with fully clonal tumors using DASH. (C-D, K-L, S-T) Line plots showing the measured (C, K, S) sensitivity and (D, L, T) specificity at various purity levels with fully clonal tumors using LOHHLA. Shaded region denotes 95% confidence intervals. (E-F, M-N, U-V) Heatmaps displaying DASH (E, M, N) sensitivity and (F, N, V) specificity to capture HLA LOH in simulated samples of differing purity and clonality for each cell line. (G-H, O-P, W-X) Heatmaps displaying LOHHLA (G, O, W) sensitivity and (H, P, X) specificity to capture HLA LOH in simulated samples of differing purity and clonality for each cell line. Dark blue denotes high sensitivity or specificity, light blue denotes low sensitivity or specificity and gray denotes no data. Source data are provided with this paper.

**Supplementary Figure 11.**


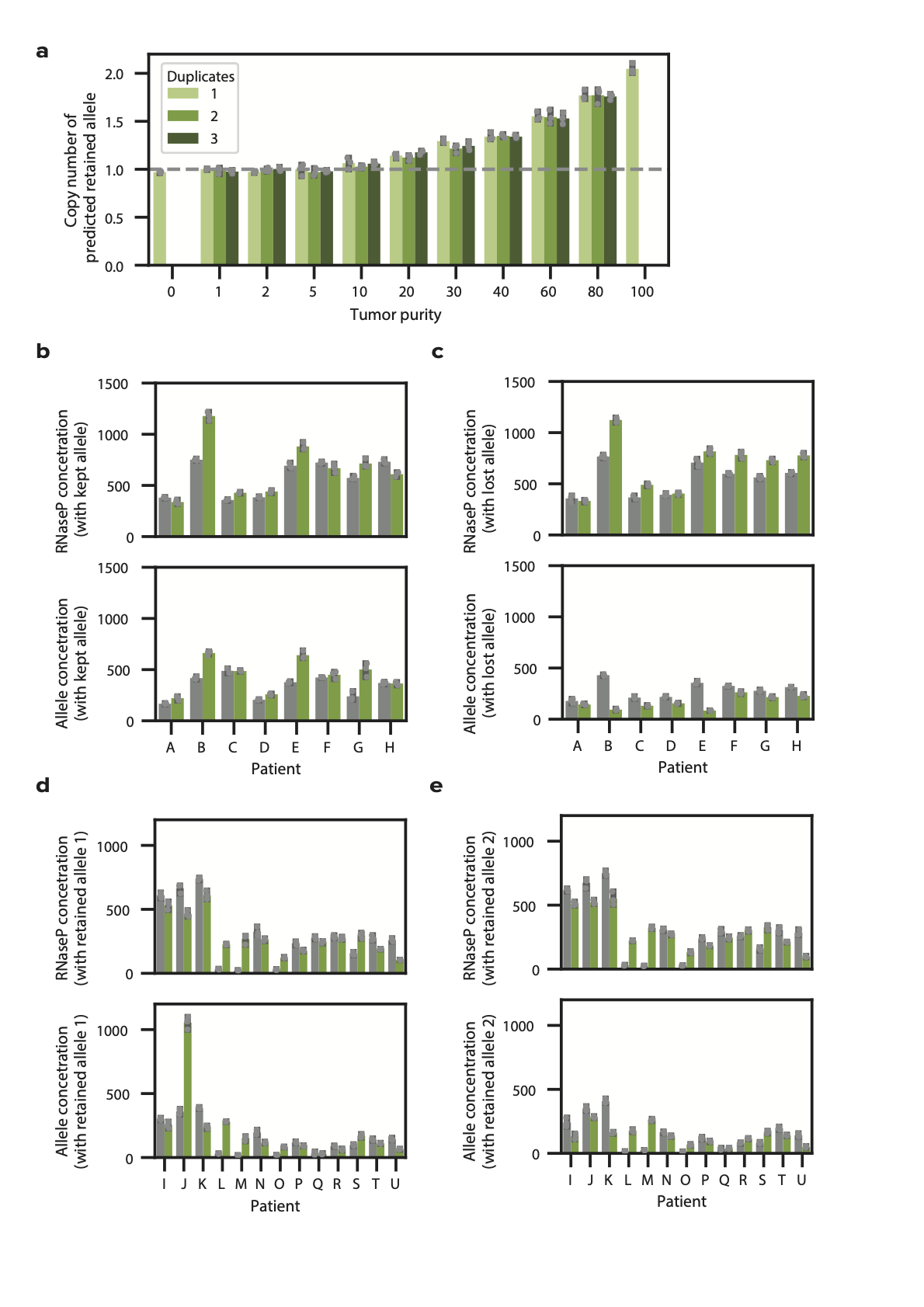


Allele-specific genomic validation with digital PCR. (A) Bar plots indicating the allele-specific copy number of the predicted kept allele, relative to *RNAse P*, as measured by dPCR for cell line mixtures of varying tumor purities, with n=3 technical replicates examined over 3 independent experiments per tumor purity. (B-C) Bar plots showing *RNAse P* and allele concentrations for normal and tumor paired samples with HLA LOH (B) with the kept allele and (C) with the lost allele, with n=3 technical replicates. (D-E) Bar plots showing *RNAse P* and allele concentrations for normal and tumor paired samples without HLA LOH (D) with the retained allele 1, (E) and with the retained allele 2, with n=3 technical replicates. 95% confidence intervals are shown in dark gray. Source data are provided with this paper.

**Supplementary Figure 12.**


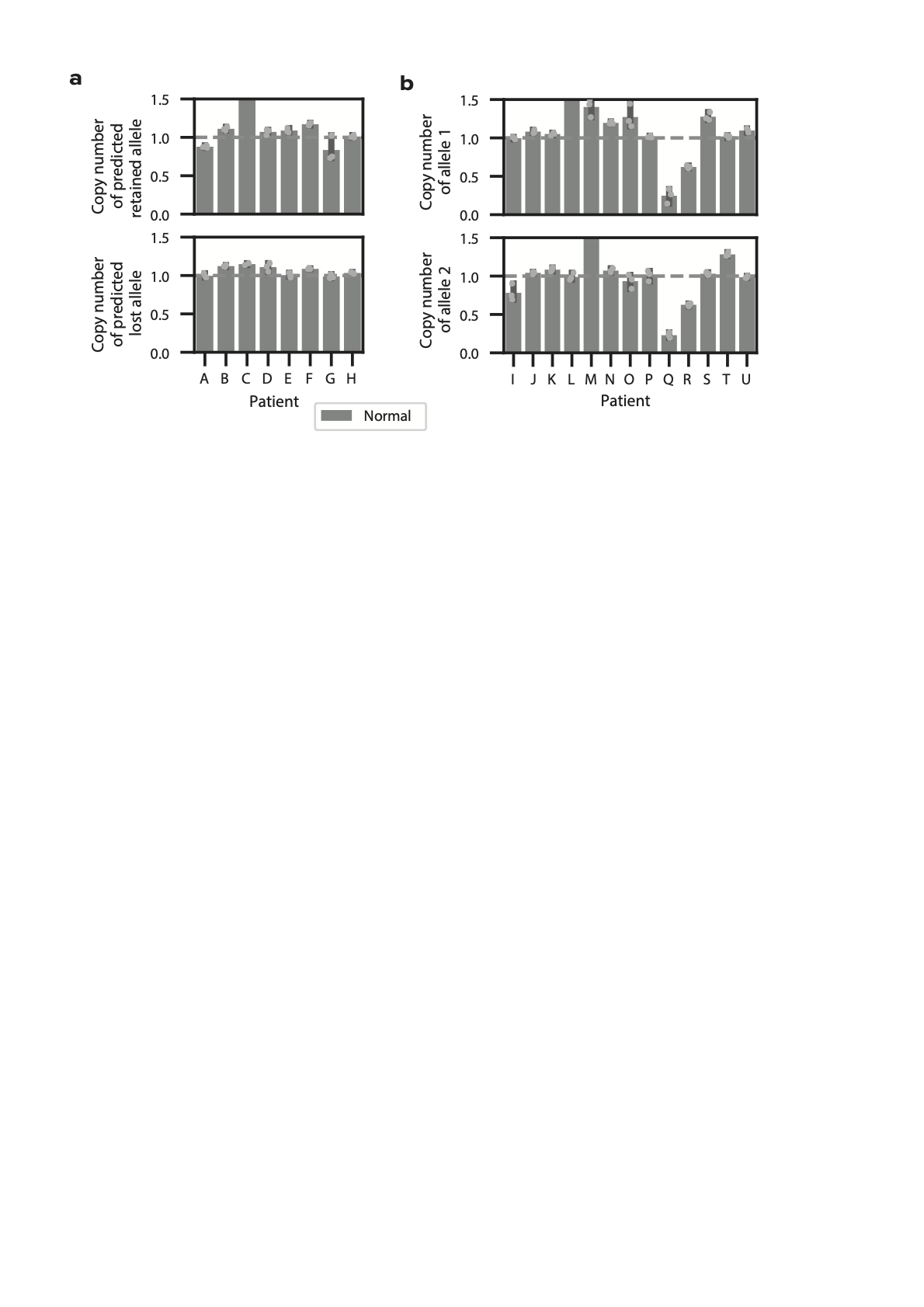


Specificities of allele-specific primers. (A) Bar plots denoting the copy numbers of both retained and lost alleles per patient using allele-specific primers in normal diploid samples, with n=3 technical replicates. (B) Bar plots denoting the copy numbers of both allele 1 and allele 2 per patient using allele-specific primers in normal diploid samples, with n=3 technical replicates. 95% confidence intervals are shown in dark gray. Source data are provided with this paper.

**Supplementary Figure 13.**


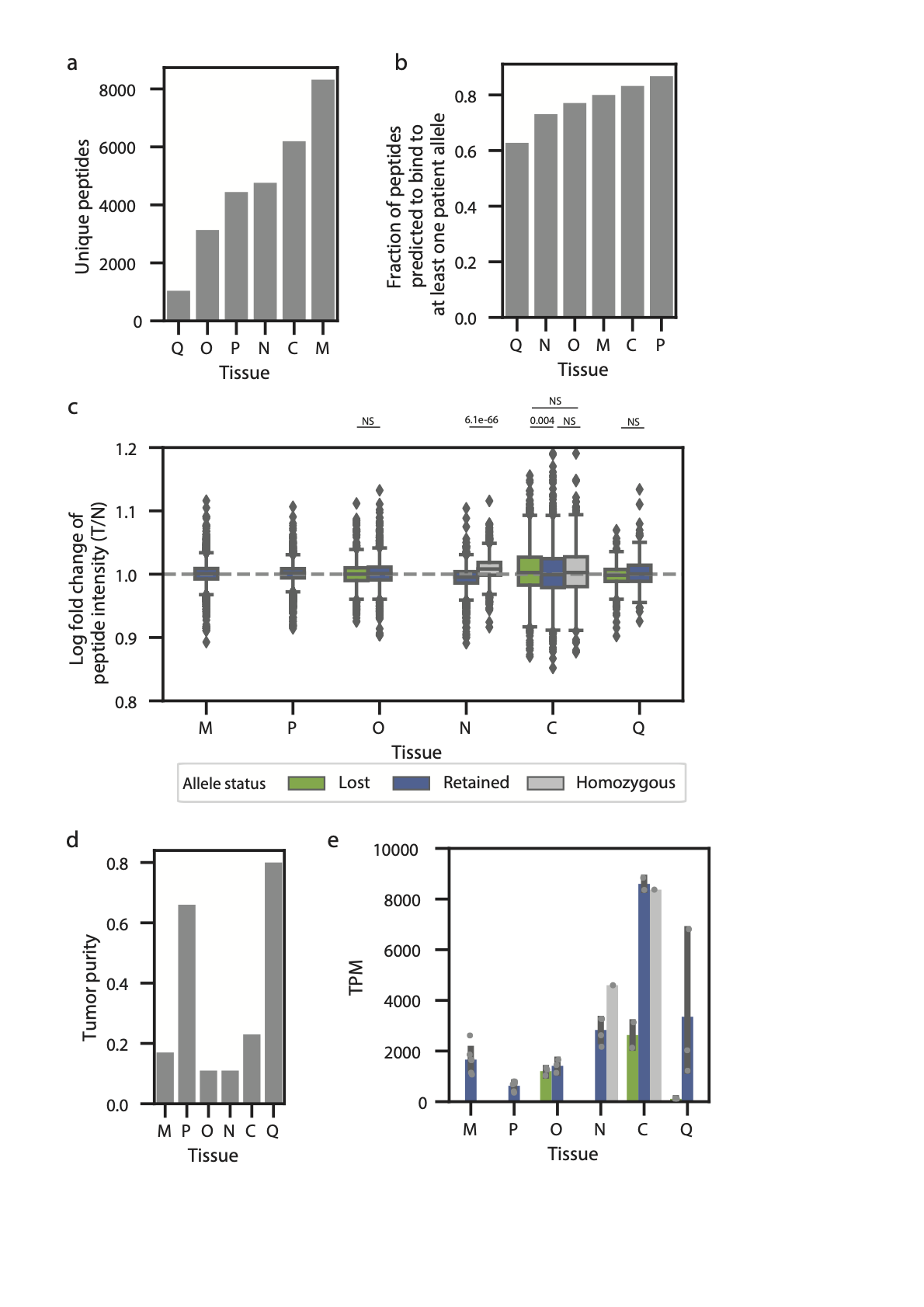


Quality and summary of quantitative immunopeptidomics data. (A) A bar plot denoting the number of unique peptides identified in each quantitative immunopeptidomics experiment. (B) A bar plot showing the fraction of peptides in each experiment that are predicted to bind to at least one of the patient’s alleles. (C) Boxplots showing the distribution of log fold peptide intensities in lost, retained and homozygous alleles for each of the six patient tissue samples. The center of the box denotes the median value, the box denotes the quartiles and the whiskers denote the remainder of the distribution apart from outliers. Statistical significance is assessed with a two-sided Student T-Test with asterisks denoting p-values less than 0.05. (D) A bar plot denoting the tumor purity of each patient tumor tissue sample. (E) Bar plots showing the allele-specific expression distribution (TPM) of lost, retained and homozygous alleles in each patient tumor tissue sample, with n ranging from one to six depending on number of alleles in each category per patient. 95% confidence intervals are shown in dark gray. Source data are provided with this paper.

**Supplementary Figure 14.**


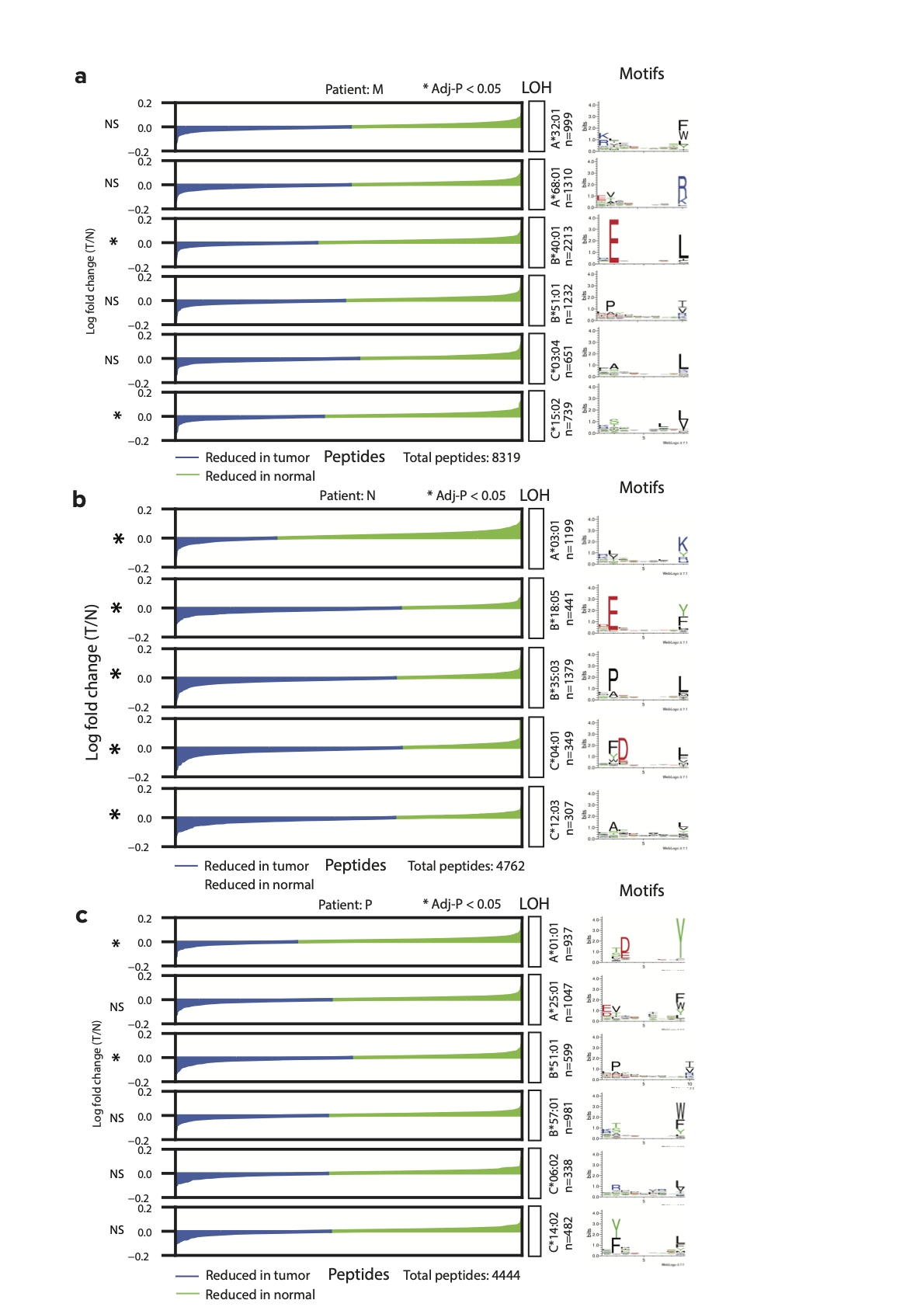


Quantitative immunopeptidomics on control samples without predicted HLA LOH. (A-B) Waterfall plots showing the log2 fold change from a normal sample to a tumor sample for peptides binding to each of the alleles in a particular patient. Blue denotes peptides that are less frequent in the tumor while green denotes peptides that are more frequent in the tumor. Gray boxes denote deleted alleles while white boxes denote retained alleles. The peptides for each allele are visualized as a motif. Statistical significance assessed using a two-sided Wilcoxon paired rank sum test. Samples shown are: (A) M, (B) N and (C) P. Source data are provided with this paper.

**Supplementary Figure 15.**

**
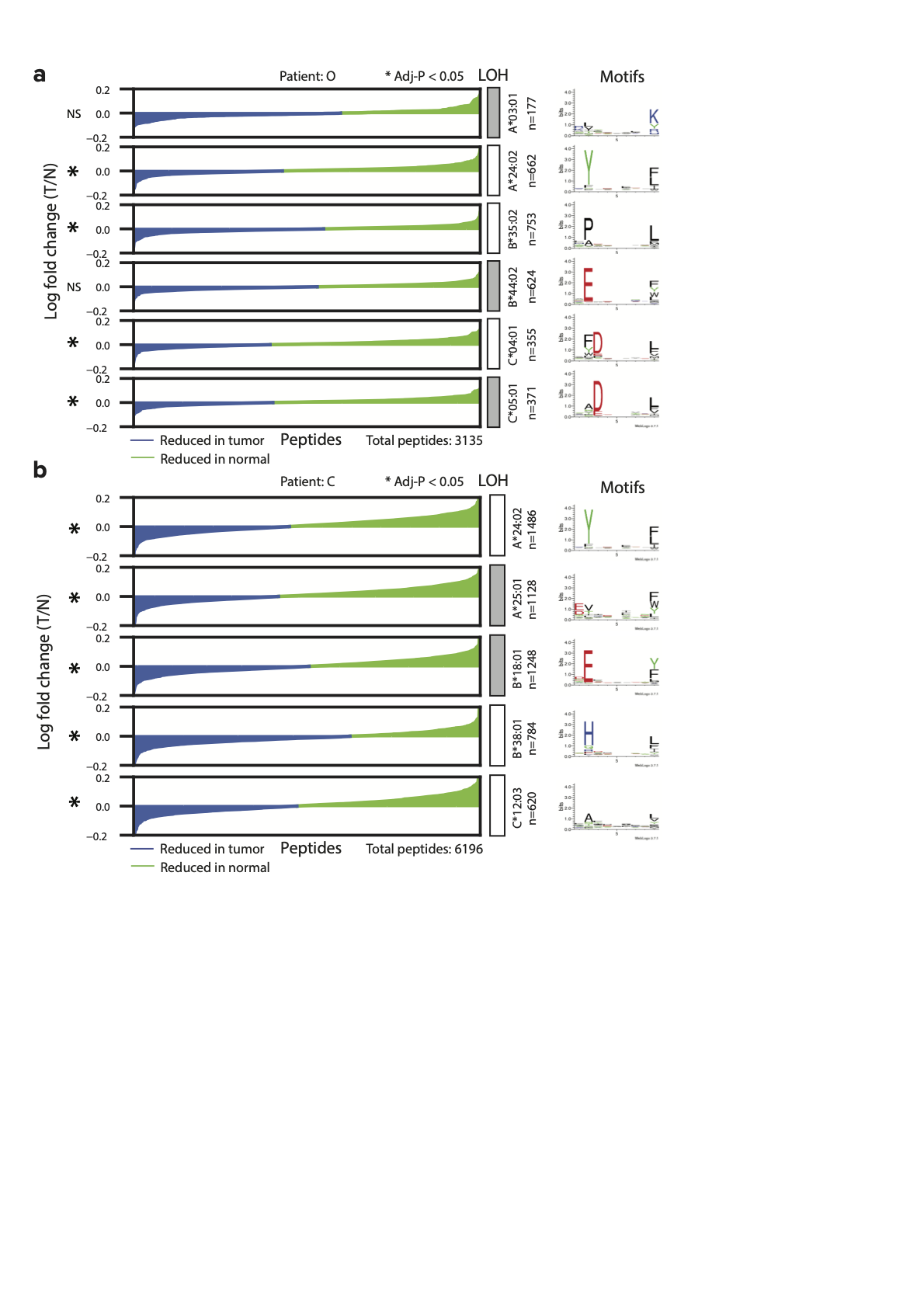
**

Quantitative immunopeptidomics on low tumor purity samples with predicted HLA LOH. (A-B) Waterfall plots showing the log2 fold change from a normal sample to a tumor sample for peptides binding to each of the alleles in a particular patient. Blue denotes peptides that are less frequent in the tumor while green denotes peptides that are more frequent in the tumor. Gray boxes denote deleted alleles while white boxes denote retained alleles. The peptides for each allele are visualized as a motif. Statistical significance assessed using a two-sided Wilcoxon paired rank sum test. Samples shown are: (A) O and (B) C. Source data are provided with this paper.

**Supplementary Figure 16.**

**
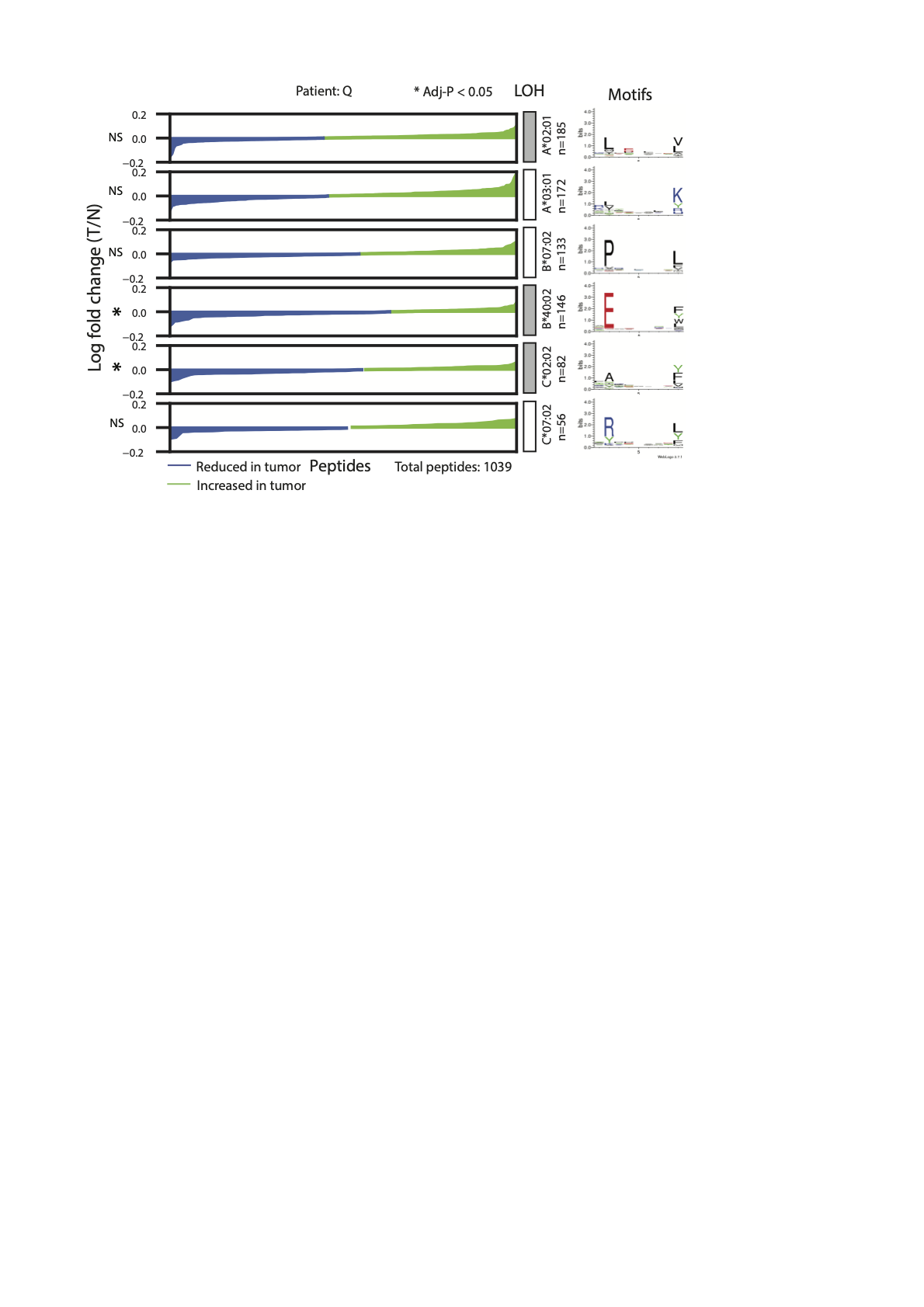
**

Quantitative immunopeptidomics on a high tumor purity sample with predicted HLA LOH. Waterfall plots showing the log2 fold change from a normal sample to a tumor sample for peptides binding to each of the alleles in a particular patient. Blue denotes peptides that are less frequent in the tumor while green denotes peptides that are more frequent in the tumor. Gray boxes denote deleted alleles while white boxes denote retained alleles. The peptides for each allele are visualized as a motif. Statistical significance assessed using a two-sided Wilcoxon paired rank sum test. Sample shown: Q. Source data are provided with this paper.

**Supplementary Figure 17.**

**
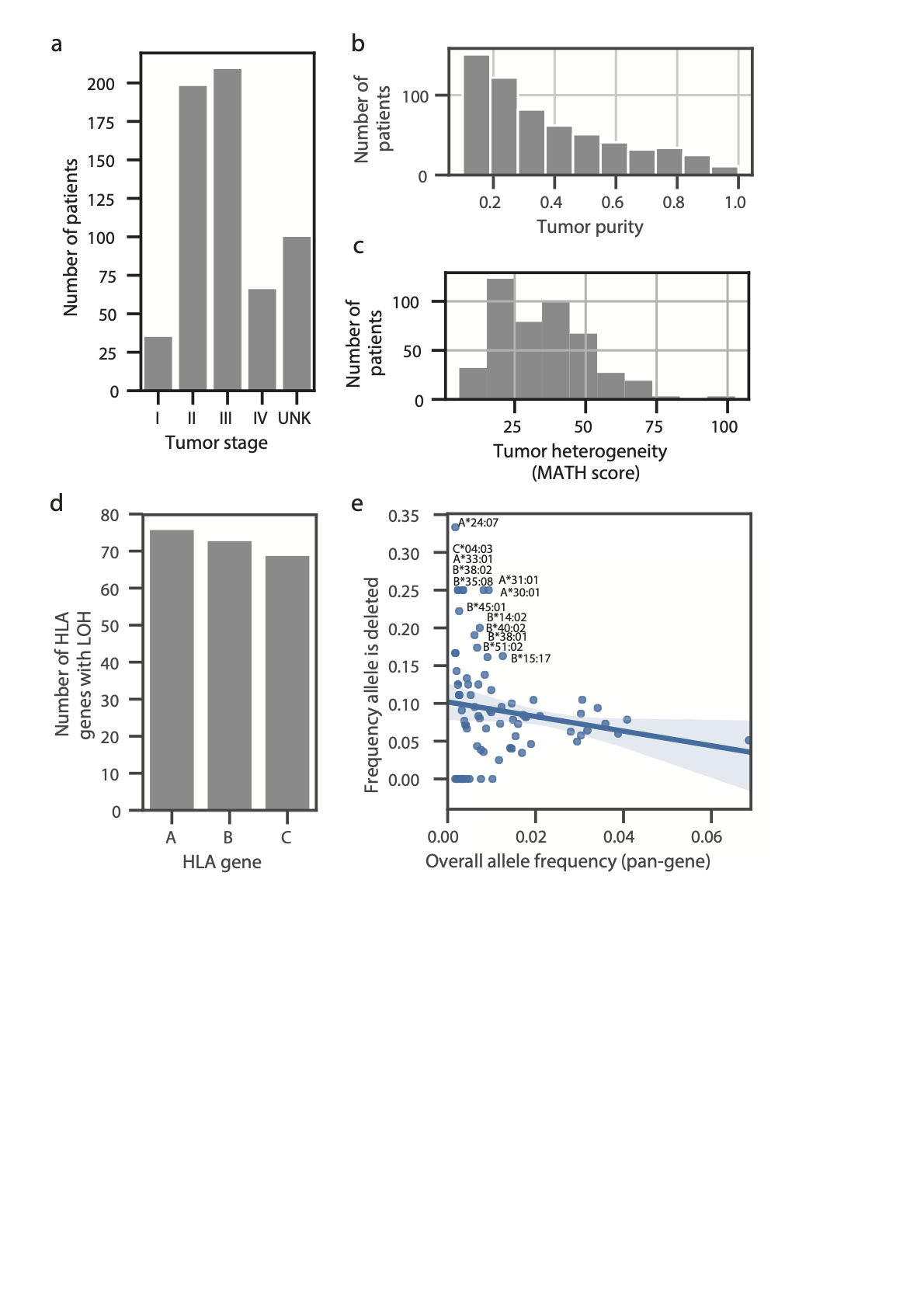
**

Tumor and allele breakdown across the patient cohort. (A-C) Histograms showing the number of patients broken down by (A) tumor stage, (B) tumor purity, and (C) tumor heterogeneity (MATH score). (D) Bar plot detailing the number of HLA LOH events present in the patient cohort broken down by gene. (E) Scatter plot showing the frequency that an allele is deleted as a function of the overall frequency that an allele appears across the cohort. The shaded region represents the 95% confidence interval. Source data are provided with this paper.

**Supplementary Figure 18.**


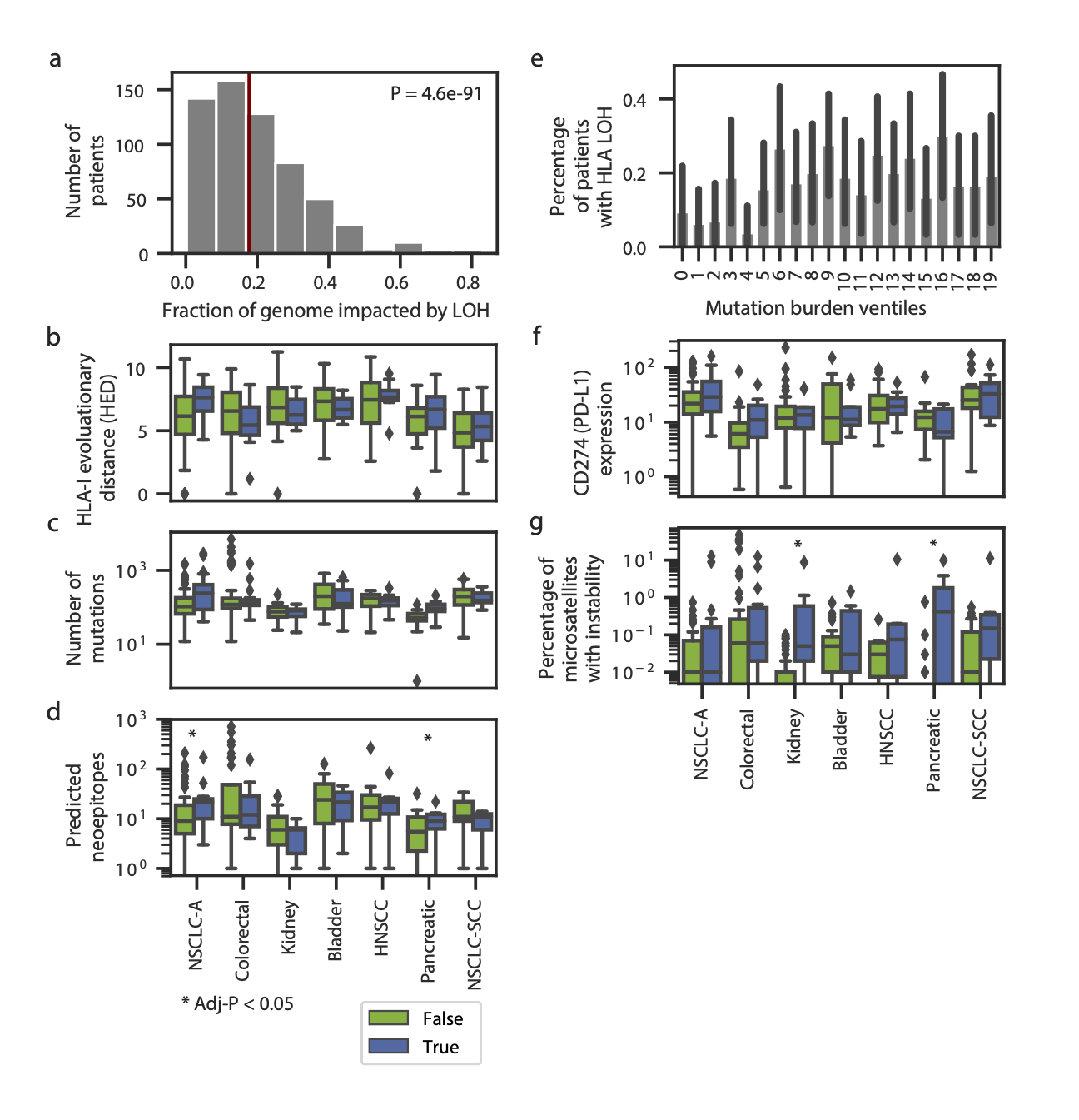


Relationship between HLA LOH and other tumor covariates. (A) Histogram of the fractions of genome-wide LOH across patients in the cohort. The red line denotes the percentage of HLA LOH in the cohort. Statistical significance was determined using a two-sided Mann Whitney U test. (B-D) Boxplots showing the distribution of (B) HLA-I Evolutionary Distance (HED), (C) number of mutations (SNV, indel and fusion) for patients with and without HLA LOH and (D) predicted neoepitopes. (E) The percentage of patients with HLA LOH in each ventile of mutation burden pan-cancer. 95% confidence intervals are shown in dark gray. (F-G) Boxplots showing the distribution of (F) CD274 (PD-L1) expression and (G) the percentage of microsatellites with instability for patients with and without HLA LOH. For B/C/D/F/G, only tumor types with at least 8 patients impacted by HLA LOH are shown (with n=88 for NSCLC-A; 80 for Colorectal; 31 for NSCLC-SCC; 55 for Kidney; 38 for Bladder; 30 for Pancreatic; 20 for HNSCC). The center of the box denotes the median value, the box denotes the quartiles and the whiskers denote the remainder of the distribution apart from outliers. Statistical analyses are performed with Mann Whitney U tests and are Bonferroni corrected. Source data are provided with this paper. NSCLC-A: Non-small cell lung cancer adenocarcinoma; NSCLC-SCC: Non-small cell lung cancer squamous cell carcinoma; HNSCC: Head and neck squamous cell carcinoma.

**Supplementary Table 1.
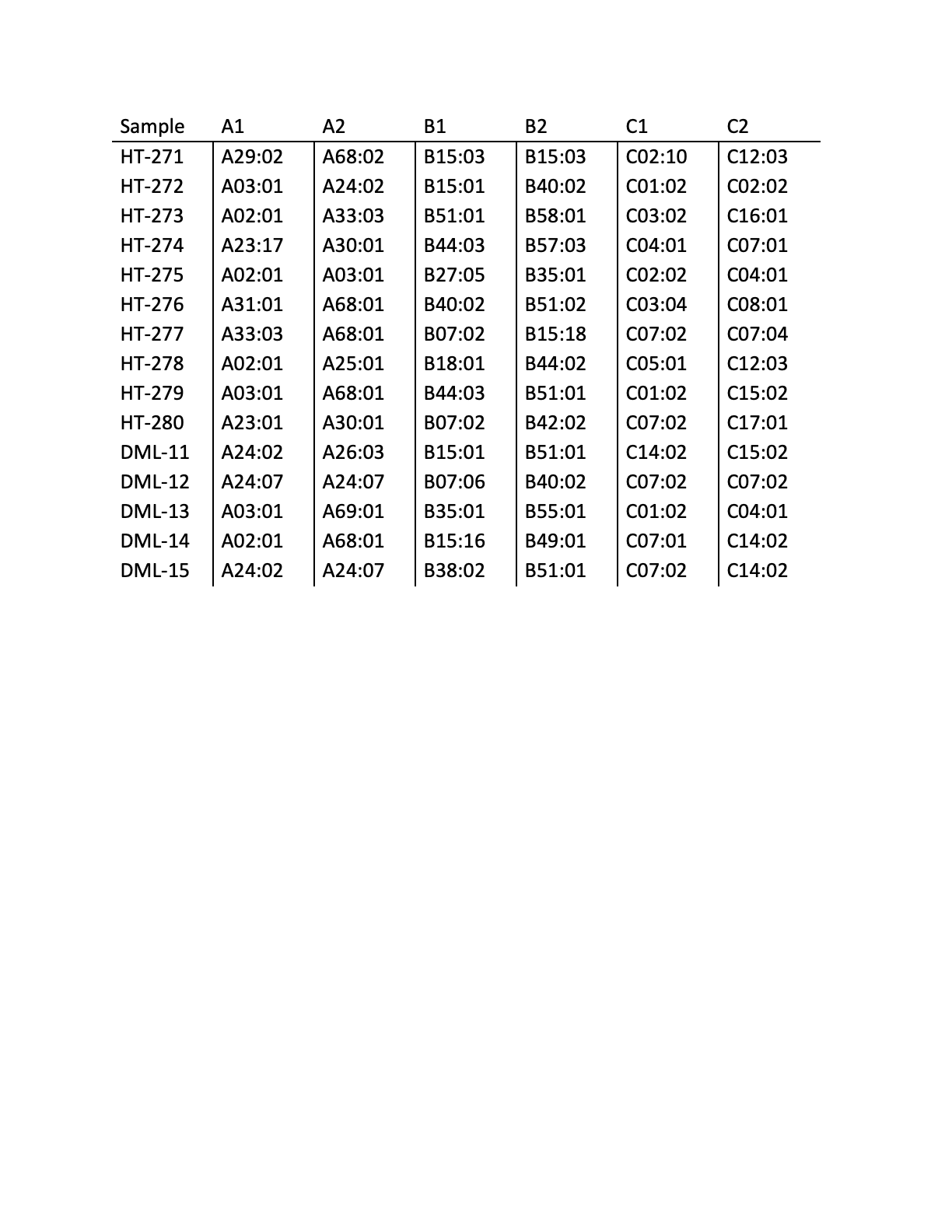
**

HLA typing validation samples. List of samples and HLA types used for HLA validation.

**Supplementary Table 2.**

**
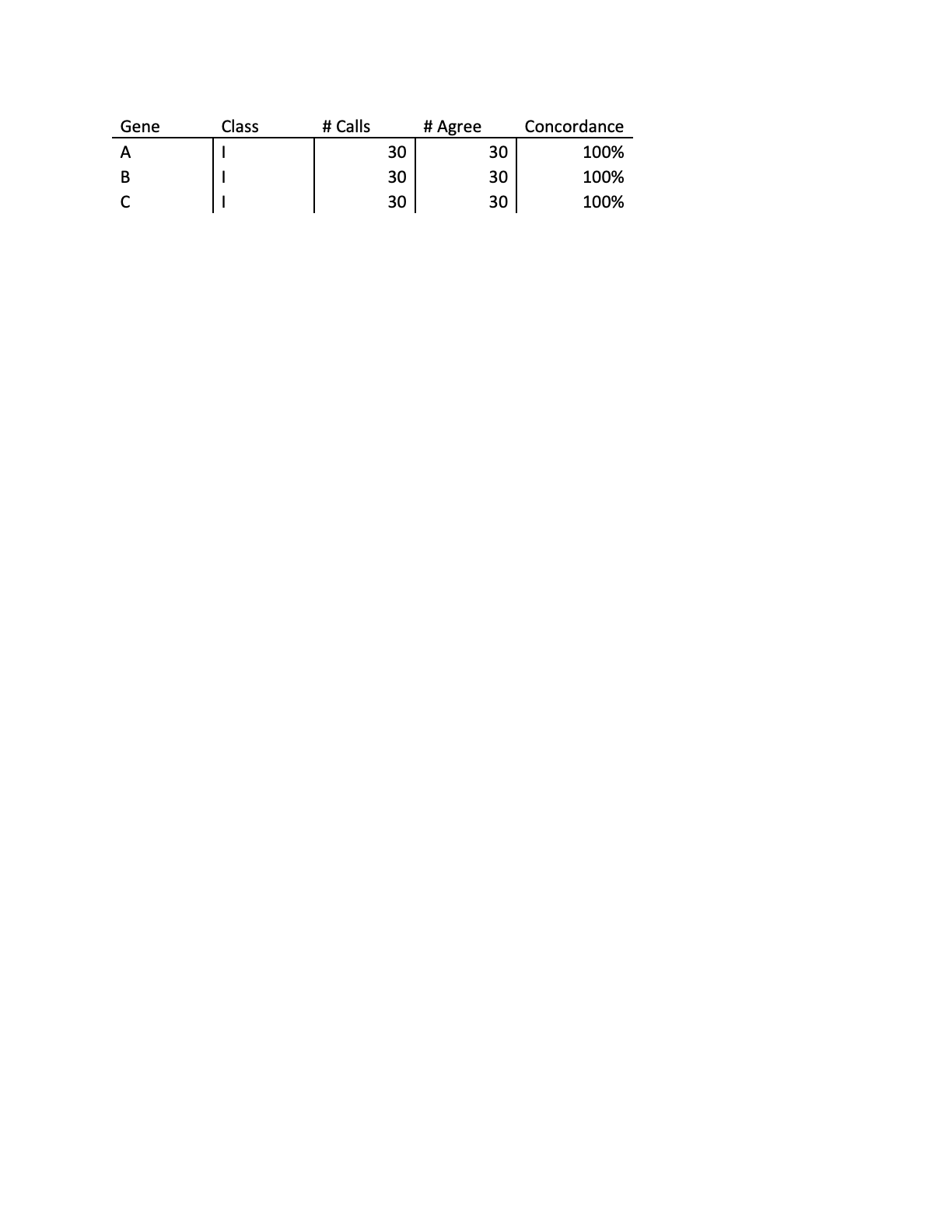
**

HLA typing validation results. Concordance results of the HLA typing experiment.

**Supplementary Table 3.**

**
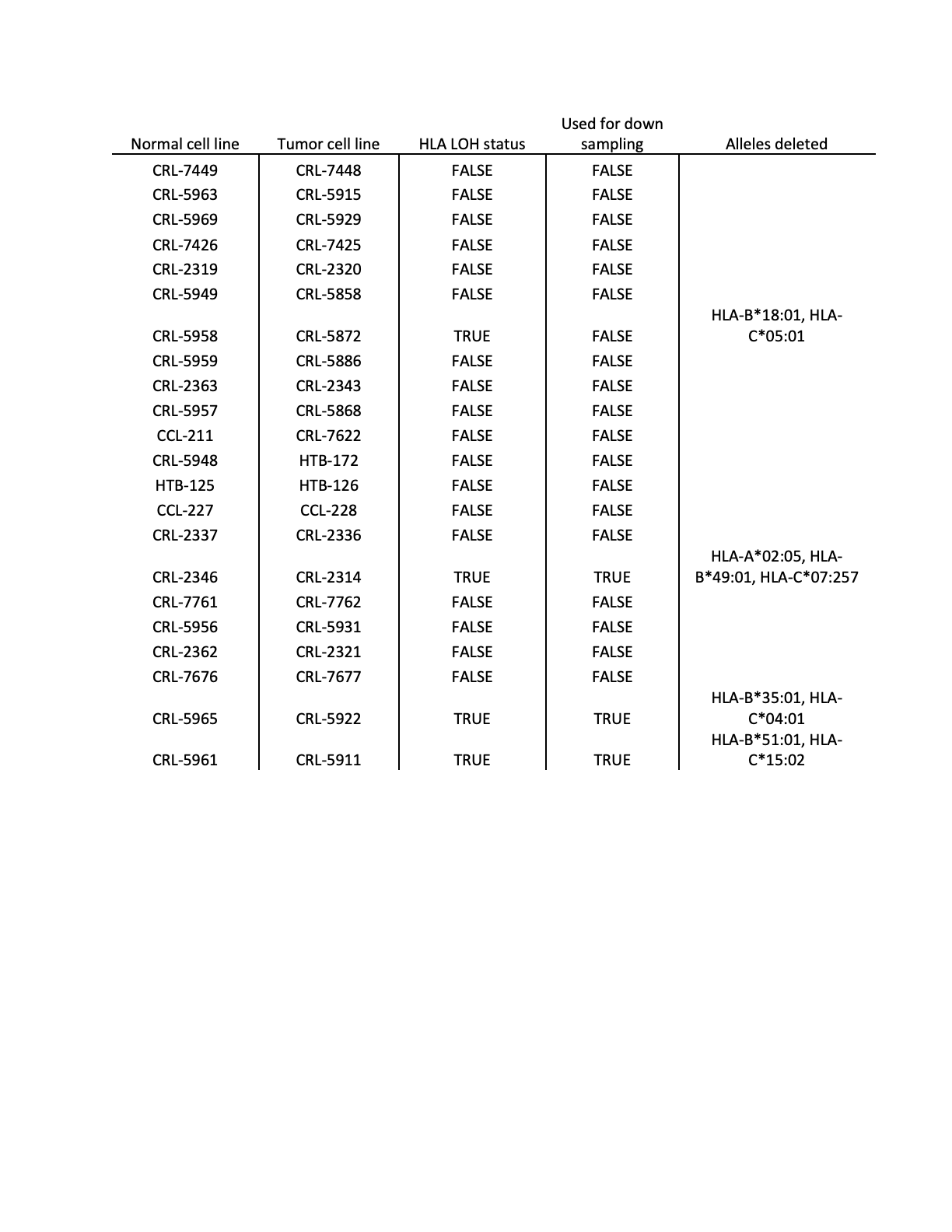
**

Cell lines profiled for HLA LOH. List of cell lines profiled by ImmunoID NeXT for HLA LOH, along with details about the cell lines used for the downsampling experiment and the specific HLA alleles that were deleted.

**Supplementary Table 4.**

**
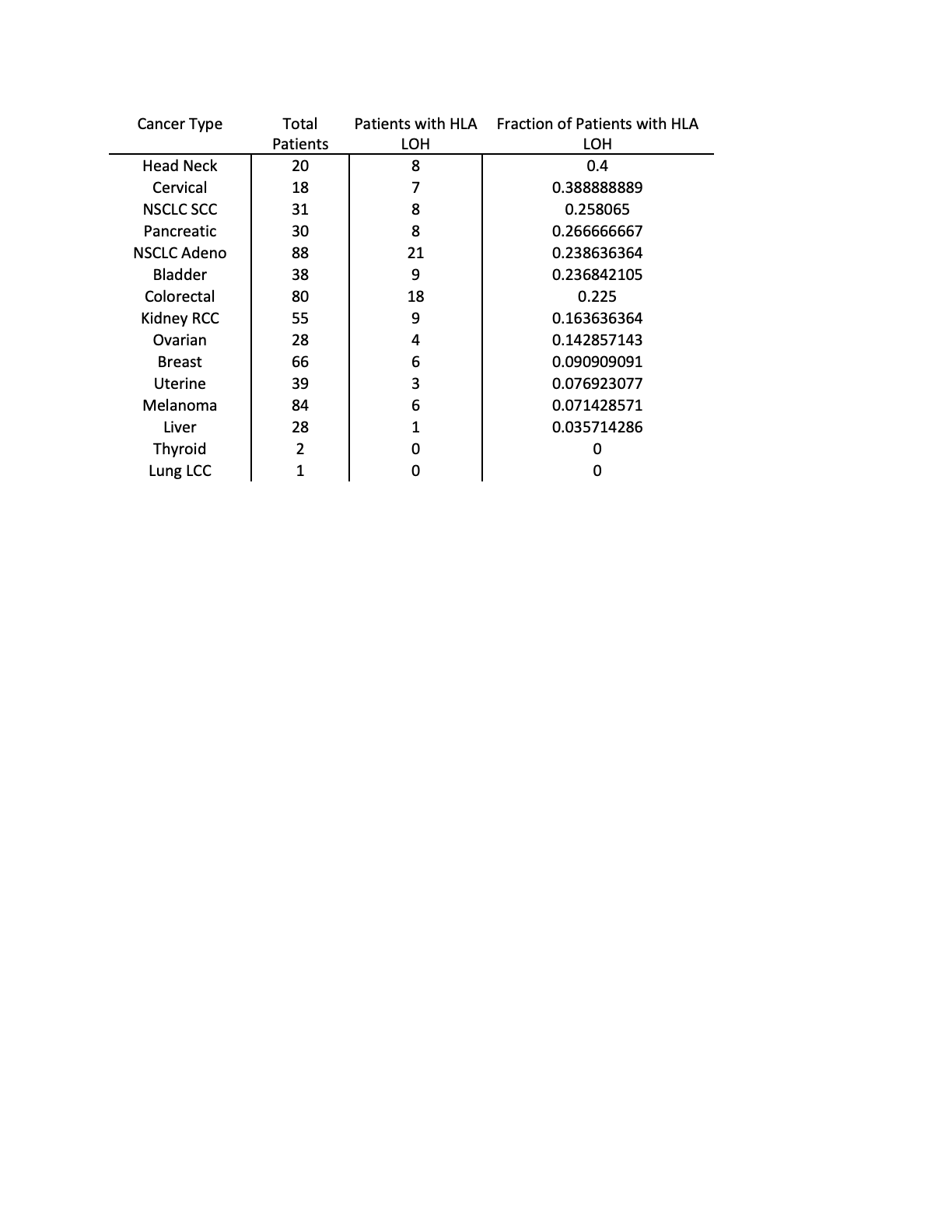
**

HLA LOH frequencies by tumor type. Total number of patients and frequency of patients with HLA LOH. Data behind Figure 5A.
