# Supplementary material for: A machine learning algorithm with subclonal sensitivity reveals widespread pan-cancer human leukocyte antigen loss of heterozygosity
Source: Nat Commun. 2022 Apr 12;13:1925. doi: 10.1038/s41467-022-29203-w (PMC9005524; doi:10.1038/s41467-022-29203-w)
Supplement: Supplementary file 3 — Description of Additional Supplementary Files [file 41467_2022_29203_MOESM3_ESM.pdf]

Title: Supplementary Data 1
Description: Cell line down sampling. Detailed overview of purity, clonality, number of normal reads, number of tumor reads, percentage of tumor reads and replicates for the cell line down sampling analysis.

Title: Supplementary Data 2
Description: Primer designs. Primer and probe sequences paired with sample information, lost gene, LOH status prediction.

Title: Supplementary Data 3
Description: Raw digital PCR results. FAM and VIC copies for each replicate and allele of each (A) cell line dilution and (B) patient.

Title: Supplementary Data 4
Description: Peptides and log fold change from quantitative immunopeptidomics. Processed quantitative immunopeptidomics data for each patient. Columns include the peptide and the log fold change intensity.

Title: Supplementary Software 1
Description: The code required to run the DASH software and to regenerate all of the figures and tables. All figures were generated using python 3.7, matplotlib 2.2.3 and seaborn 0.9.0.
