# Supplementary material for: A machine learning algorithm with subclonal sensitivity reveals widespread pan-cancer human leukocyte antigen loss of heterozygosity
Source: Nat Commun. 2022 Apr 12;13:1925. doi: 10.1038/s41467-022-29203-w (PMC9005524; doi:10.1038/s41467-022-29203-w)
Supplement: Supplementary file 8 — Supplementary Software [file 41467_2022_29203_MOESM8_ESM.zip › Manuscript_code.2022_03_14/LICENSE]

Personalis DASH Non-Commercial Software License Agreement

This license agreement ("License"), effective today, is made by and between you ("Licensee") and Personalis Inc., a Delaware corporation having its offices at 1330 O'Brien Drive, Menlo Park, CA 94025 ("Personalis") and concerns certain software known as "DASH (**D**eletion of **A**llele-**S**pecific **H**LAs)," a machine learning algorithm to detect HLA LOH, developed by Personalis and includes executable code, source code, and documentation ("Software").

1. **General.** A limited, non-exclusive, nontransferable, perpetual license is granted to the Licensee to install and use the Software for academic, non-profit, or government-sponsored research purposes. Use of the Software under this License is restricted to non-commercial purposes. Commercial use of the Software requires a separately executed license agreement.
2. **Permitted Use and Restrictions.** Licensee agrees that it will use the Software, and any modifications, improvements, or derivatives to the Software that the Licensee may create (collectively, "Improvements") solely for internal, non-commercial purposes and shall not distribute or transfer the Software or Improvements to any person or third parties without prior written permission from Personalis. The term "non-commercial," as used in this License, means academic or other scholarly research which (a) is not undertaken for profit, or (b) is not intended to produce works, services, or data for commercial use, or (c) is neither conducted, nor funded, by a person or an entity engaged in the commercial use, application or exploitation of works similar to the Software.
3. **Ownership and Assignment of Copyright.** The Licensee acknowledges that Personalis holds copyright in the Software and associated documentation, and the Software and associated documentation are the property of Personalis. The Licensee agrees that any Improvements made by Licensee shall be subject to the same terms and conditions as the Software. Licensee agrees not to assert a claim of infringement in Licensee copyrights in Improvements in the event Personalis prepares substantially similar modifications or derivative works. The Licensee agrees to use his/her reasonable best efforts to protect the contents of the Software and to prevent unauthorized disclosure by its agents, officers, employees, and consultants. If the Licensee receives a request to furnish all or any portion of the Software to a third party, Licensee will not fulfill such a request but will refer the third party to Personalis so that the third party's use of this Software will be subject to the terms and conditions of this License. Notwithstanding the above, Licensee may disclose any Improvements that do not involve disclosure of the Software.
4. **Copies.** The Licensee may make a reasonable number of copies of the Software for the purposes of backup, maintenance of the Software or the development of derivative works based on the Software. These additional copies shall carry the copyright notice and shall be controlled by this License, and will be destroyed along with the original by the Licensee upon termination of the License.

5. **Acknowledgement.** Licensee agrees that any publication of results obtained with the Software will acknowledge its use by an appropriate citation as specified in the documentation.
6. **Disclaimer of Warranties and Limitation of Liability.** THE LICENSEE AGREES THAT ANY EXPRESS OR IMPLIED WARRANTIES, INCLUDING, BUT NOT LIMITED TO, THE IMPLIED WARRANTIES OF MERCHANTABILITY AND FITNESS FOR A PARTICULAR PURPOSE ARE DISCLAIMED. PERSONALIS MAKES NO REPRESENTATION OR WARRANTY THAT THE SOFTWARE WILL NOT INFRINGE ANY PATENT OR OTHER PROPRIETARY RIGHT. IN NO EVENT SHALL PERSONALIS BE LIABLE FOR ANY DIRECT, INDIRECT, INCIDENTAL, SPECIAL, EXEMPLARY, OR CONSEQUENTIAL DAMAGES (INCLUDING, BUT NOT LIMITED TO, PROCUREMENT OF SUBSTITUTE GOODS OR SERVICES; LOSS OF USE, DATA, OR PROFITS; OR BUSINESS INTERRUPTION) HOWEVER CAUSED AND ON ANY THEORY OF LIABILITY, WHETHER IN CONTRACT, STRICT LIABILITY, OR TORT (INCLUDING NEGLIGENCE OR OTHERWISE) ARISING IN ANY WAY OUT OF THE USE OF THIS SOFTWARE, EVEN IF ADVISED OF THE POSSIBILITY OF SUCH DAMAGE.
7. **Indemnification.** To the extent permitted by law, Licensee shall indemnify, defend with counsel acceptable to Personalis, and hold harmless Personalis and the developers of the Software, their successors, agents, officers, and employees against any and all claims, liability, cost, damage, deficiency, loss, or obligation, of any kind or nature (including, without limitation, reasonable attorney’s fees and other costs and expenses of defense), including without limitation any cause of action relating to product liability, any incidental or consequential damage either direct or indirect, whether incurred, made or suffered by Licensee or any third party obtaining access to the Software through Licensee, in connection with and to the extent arising out of, the Licensee’s furnishing, performance, possession or use of the Software or in connection with the Licensee’s exercise of its rights under this License. This provision shall survive termination of this License.
8. **Termination.** This License is effective until terminated by either party. Licensee's rights under this License will terminate automatically without notice from Personalis if Licensee fails to comply with any term(s) of this License. Licensee may terminate the License by giving written notice of termination to Personalis. Upon termination of this License, Licensee shall immediately discontinue all use of the Software and destroy the original and all copies, full or partial, of the Software, including any modifications or derivative works, and associated documentation.
9. **Use of Names and Trademarks.** Nothing contained in this License will be construed as conferring any right to either party to use in advertising, publicity, or other promotional activities any name, trade name, trademark, or other designation of the other party (including a contraction, abbreviation or simulation of any of the foregoing). The use by the Licensee of the name "Personalis" or “DASH” in advertising, publicity or other promotional activities is expressly prohibited.

10. **Governing Law and General Provisions.** This License shall be governed by the laws of the State of California, excluding the application of its conflicts of law rules. This License shall not be governed by the United Nations Convention on Contracts for the International Sale of Goods, the application of which is expressly excluded. If any provisions of this License are held invalid or unenforceable for any reason, the remaining provisions shall remain in full force and effect. This License is binding upon any heirs and assigns of the Licensee. The License granted to Licensee hereunder may not be assigned or transferred to any other person or entity without the express consent of Personalis. This License constitutes the entire agreement between the parties with respect to the use of the Software licensed hereunder and supersedes all other previous or contemporaneous agreements or understandings between the parties, whether verbal or written, concerning the subject matter. Any translation of this License is done for local requirements and in the event of a dispute between the English and any non-English versions, the English version of this License shall govern.
